# Molecular of *Anaplasma marginale* Theiler (Rickettsiales: Anaplasmataceae) in horseflies (Diptera: Tabanidae) in Uruguay

**DOI:** 10.1038/s41598-022-27067-0

**Published:** 2022-12-28

**Authors:** Gratchela D. Rodrigues, Martín Lucas, Hadassa Gabriela Ortiz, Laís dos Santos Gonçalves, Eduardo Blodorn, William Borges Domingues, Leandro Silva Nunes, Anderson Saravia, Pablo Parodi, Franklin Riet-Correa, Alejo Menchaca, Vinicius Farias Campos, Tiago Kütter Krolow, Rodrigo Ferreira Krüger

**Affiliations:** 1 Ecology of Parasites and Vectors Group, Universidade Federal de Pelotas, Rio Grande do Sul, Brazil; 2 Instituto Nacional de Investigación Agropecuaria (INIA), Plataforma de Investigación en Salud Animal, Tacuarembó, Uruguay; 3 Structural Genomics Group, Centro de Desenvolvimento Tecnológico, Universidade Federal de Pelotas, Rio Grande do Sul, Brazil; 4 Programa de Pós-Graduação em BiodiversidadeEcologia e Conservação (PPGBEC), Universidade Federal do Tocantins, Tocantins, Brazil

**Keywords:** Molecular biology, Parasitology, Pathogens

## Abstract

*Anaplasma marginale* is transmitted biologically by infected ticks or mechanically by biting flies and contaminated fomites. In tick-free areas, such as southern Uruguay, horseflies could be the principal vectors of this pathogen for bovines, causing anaplasmosis. The objective of this work was to detect the presence of *A. marginale* by MSP-5 PCR and Sanger sequencing in the most prevalent species of horseflies obtained using different collection methods in Colonia, Tacuarembó and Paysandú, Uruguay. Eight horsefly species were tested (*Dasybasis missionum*, *Poeciloderas lindneri, Tabanus campestris*, *T. claripennis, T. fuscofasciatus, T. platensis*, *T. tacuaremboensis* and *T. triangulum*); four species were found to be positive for *A. marginale*, with *D. missionum* and *P. lindneri* having the most frequent infections, while only one individual each of *T. fuscofasciatus* and *T. tacuaremboensis* was positive. Both *D. missionum* and *P. lindneri* were positive for *A. marginale* in tick-free areas, and the implications are discussed in this report.

## Introduction

*Anaplasma marginale* Theiler, 1910 (Rickettsiales: Anaplasmataceae) is an intracellular pathogen endemic to tropical and subtropical areas worldwide. Infection of cattle with *A. marginale* causes bovine anaplasmosis, a mild to severe haemolytic disease that results in considerable economic losses to both the dairy and beef industries. Transmission of *A. marginale* to cattle occurs biologically by ticks and mechanically by biting flies and by blood-contaminated fomites^1^. In recent studies, biological transmission by ticks was reported to be more efficient than mechanical transmission by *Stomoxys calcitrans* Linnaeus, 1758 (Diptera: Muscidae), the stable fly^2^, and the horsefly *Tabanus fuscicostatus* Hine, 1906 (Diptera: Tabanidae) in the southeastern United States^3^. Despite this, mechanical transmission by horseflies is considered important to anaplasmosis epidemiology^4–6^. In tick-free areas where anaplasmosis outbreaks are often detected, transmission by flies and fomites deserves further investigation^3,5,7^.

Uruguay is located between latitudes 30° and 35°S and longitudes 53° and 58°W, has a temperate climate and is considered marginal for the development of cattle ticks^8^. The country has a bovine population that exceeded 11.8 million animals in 2021^9^ in two areas with the occurrence of *Rhipicephalus *(*Boophilus*)* microplus* (Canestrini, 1888) (Ixodida, Ixodidae), a biological vector of *A. marginale*. The northern area of the Rio Negro is considered tick infested, and the southern area has variable infestations with a tick-free area^10^. This generates enzootic instability in a herd. Due to this instability, tick-borne diseases are widely distributed in the Uruguayan territory and cause substantial economic losses in the country due to the cost of control measures and animal losses^11–13^.

There is no information on the potential of horseflies to carry *A. marginale* in tick-free areas or in infested areas. The Tabanidae diversity in Uruguay comprises 46 species in 14 genera^14,15^ and allows us to infer that some species may be positive for this pathogenic agent and that, therefore, they have great epidemiological importance in anaplasmosis as well as in bovine parasitic sadness. The importance of horseflies can be even greater in locations where there is no occurrence of the biological vector, where there was no introduction of animals from areas with high infestation of ticks or where there was no possibility of fomites contacting animals on a property. Therefore, the objective of this work was to detect the presence of *A. marginale* DNA by PCR and DNA sequencing in the most prevalent species of horseflies using manual (feeding on cattle) or NZI trap collection methods in tick-free areas and infested areas in Uruguay.

## Results

**Figure 1:**
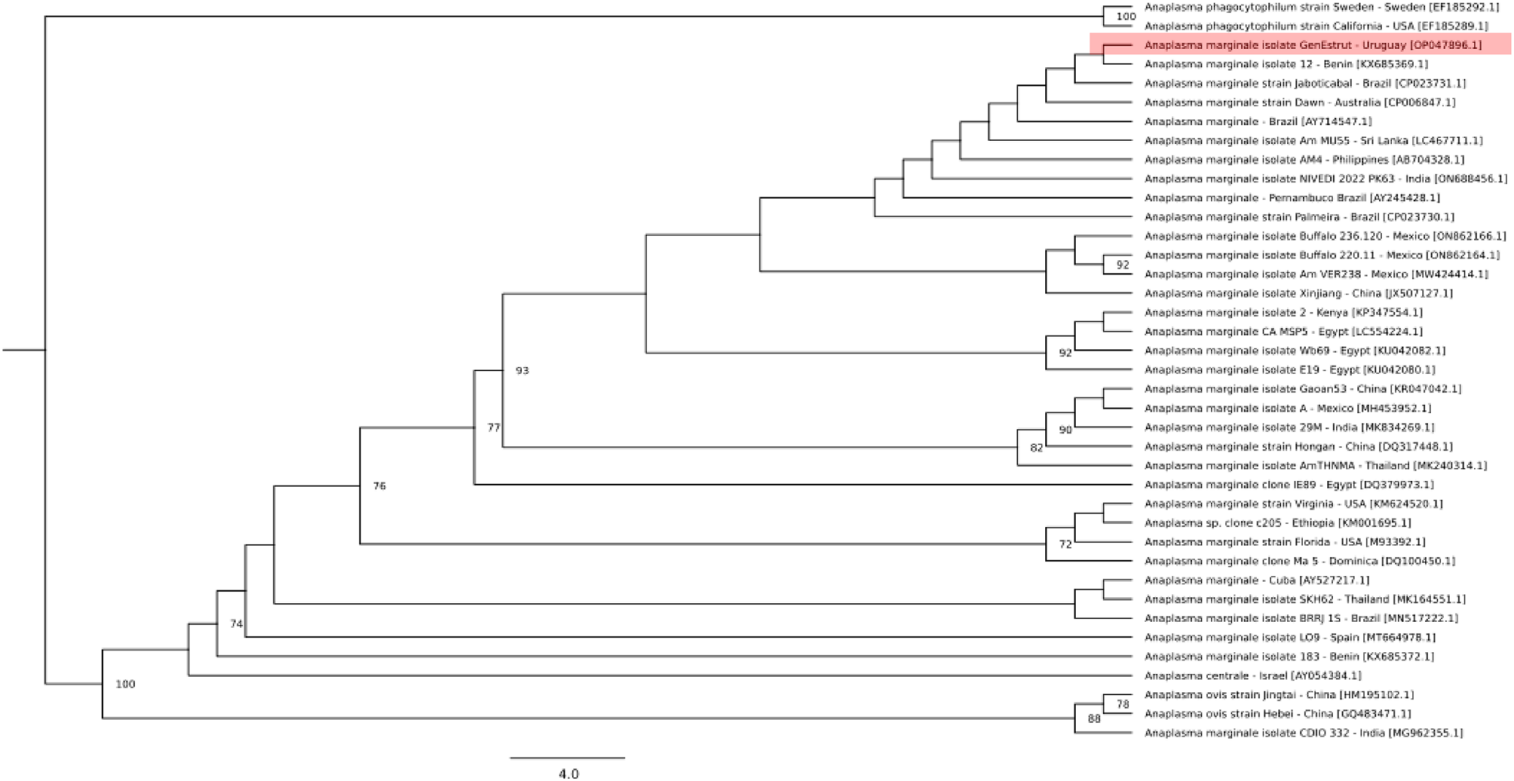
Characterization of the *A. marginale* partial *msp5* sequence. The tree was constructed using the maximum likelihood method with the evolutionary model K2P + G4. The numbers in the tree indicate bootstrap values for the branch nodes. Sequences of *A. phagocytophilum* were used as outgroups.

The GenBank search along with our sequences resulted in a final dataset of 39 sequences from 19 countries. The *msp5* sequences obtained from GenBank varied in length (351–1146 nt), and after alignment and removal of ambiguous regions with Gblocks, resulted in an alignment of 341 nt, of which 122 nt were parsimony-informative and 193 nt were constant sites. The best-fitting evolutionary model indicated by ModelFinder (according to the Bayesian information criterion (BIC)) was K2P + G4. The GenEstrut isolate was grouped with strains and isolates of *A. marginale* from Brazil, Benin, Australia, Sri Lanka, the Philippines, India, Mexico, China, Kenya, Egypt and Thailand with 93% bootstrap support (Fig. 1).

### Frequency

According to visualization of the electrophoresis gel, the molecular weight of the gene amplified by PCR was approximately 354 bp. The gene sequence is available from GenBank under accession OP047896. PCR for the MSP-5 gene indicated the presence of *A. marginale* in 26 (26.8%) of 98 specimens of tabanids. A higher prevalence was observed in *P. lindneri,* with 85% PCR positivity. The second highest prevalence was detected in *D. missionum*, at 26%, followed by *T. fuscofasciatus* and *T. tacuaremboensis*, with just one positive case each (Table 1). *T. campestri*s, *T. claripennis*, *T.* aff. *platensis* and *T. triangulum* were not PCR positive for *A. marginale* (Table 1).

**Table 1:** Species and total specimens of Tabanidae collected using different methods to detect *A. marginale* by PCR and Sanger sequencing in the departments of Tacuarembó, Paysandú and Colonia, Uruguay. M, manually; NZi, NZi trap. Number of individuals (positive individuals).

Species	Tacuarembó	Paysandú		Colonia		Total
	Methods					
	M	M	NZi	M	NZi	
	Number of individuals (Positive individuals)					
*D. missionum* (Macquart), 1838	7 (5)	0	0	0	15 (1)	22 (6)
*P. lindneri* (Kröber), 1929	0	3 (2)	11 (11)	4 (3)	2 (2)	20 (18)
*T.* aff. *platensis* Brèthes, 1910	0	0	2	0	1	3
*T. campestris* Brèthes, 1910	0	0	1	0	0	1
*T. claripennis* (Bigot), 1892	0	0	0	0	2	2
*T. fuscofasciatus* Macquart, 1838	0	14 (1)	0	4	0	18 (1)
*T. triangulum* Wiedemann, 1828	0	0	0	0	21	21
*T. tacuaremboensis* Krolow, Lucas & Henriques, 2022	0	1 (1)	0	0	8	9 (1)
Total	7 (5)	18 (4)	14 (11)	8 (3)	49 (3)	96 (26)

More MSP-5 PCR-positive tabanids were detected with manual sampling from animals being fed upon (68.8%) than with NZI traps (18.3%), independent of the location (Table 1).

The *A. marginale*-positive specimens were more proportionally abundant at Taquarembó, with 71% of *D. missionum* individuals positive, followed by Paysandu, with 47%, and Colonia, with 10% of individuals positive for *A. marginale*. At the three locations with manual collections, 75% positive individuals were obtained in Tacuarembó, 37% were positive for *A. marginale* in Colonia, and 22% were positive in Paysandú. Regarding the collections with NZI traps, Paysandú included 78% of the individuals positive for *A. marginale*, while in Colonia, the percentage of positive individuals was 6.1%.

## Discussion

This is the first study on the molecular detection of *A. marginale* in horseflies in South America and the first record of this pathogen in the species *D. missionum*, *P. lindneri* and *T. fuscofasciatus*. These three species join a list of more than 30 species in which *A. marginale* has already been detected. This represents the first occurrence of this parasite in the *Poeciloderas* genus and the second occurrence in a species of *Dasybasis*. In the genus *Tabanus*, *A. marginale* has already been detected in approximately 20 species^4–6^.

Another important result is the occurrence of horsefly specimens positive for *A. marginale* in areas free of *R. microplus* ticks, the main vector of this pathogen in Uruguay. On farms in the department of Colonia, tests were carried out on seven species of horsefly with high abundance that were collected in these areas, and positive specimens were found among *P. lindneri*, the species most frequently collected in the area^15^, and *D. missionum*.

Even though horseflies do not have the same vectorial capacity as ticks in transmitting *A. marginale*, it is worth noting that transmission factors may also depend on the abundance of these flies in the environment, as well as on tick biology, particularly the capacity to move from one host to another. This capacity is considered normal for 3-host ticks, such as the rocky mountain tick *Dermacentor andersoni* Stiles, 1908^3^, but it may be limited or very restricted in one-host ticks such as *R. microplus*^6^. In such cases, the objective of the tick is to multiply and reinject parasites on the same animal. This amplifies the parasite burden, leading to immune failure and the appearance of clinical signs. In contrast, biting flies can transmit a small quantity of blood, acting as mechanical vectors of *Anaplasma*, which is responsible for epizootics, especially in areas without efficient biological vectors^6^, such as tick-free areas.

Until now, the role of blood-sucking flies in the transmission of *A. marginale* has been poorly understood, despite outbreaks of anaplasmosis occurring in tick-free areas. In the absence of substantiating evidence regarding the transmission of *Anaplasma* in tick-free areas, the relationship between anaplasmosis outbreaks and a high abundance of horseflies has indicated that these factors are related^7,16^, but this has not been effectively proven^17^. However, in tick-free areas of Argentina, anaplasmosis outbreaks occur for unknown reasons at 4- to 7-year intervals^18^. In tick-free areas, the occurrence of anaplasmosis outbreaks on farms where there were no previously infected animals or contaminated fomites draws attention to haematophagous dipterans, such as horseflies and stable flies, as potential mechanical vectors^7,19^. From this perspective, our results point to *P. lindneri* and *D. missionum* as potential vectors of this pathogen.

Our point of view on the importance of horseflies in the epidemiology of anaplasmosis in Uruguay and especially in tick-free areas has arisen because samples collected from organisms on animals showed a higher prevalence of *Anaplasma* than those collected with NZI traps. The contact between horseflies and animals can increase the probability of occurrence of *Anaplasma* in vectors, as observed in this work. Thus, mechanical transmission is likely the major route of dissemination for *A. marginale* in certain areas of the USA^1^, Central and South America and Africa where tick vectors are absent^4,20^. In addition, special attention should be given to potential reservoirs of *A. marginale*, which could serve as a source of infective blood for mechanical spread by various routes and biological transmission by ticks^1^. These are perhaps the first animals to suffer haematophagy by horseflies and can mechanically contaminate the flies (see Figure 3 in De La Fuente et al.^16^).

The species *D. missionum* and *P. lindneri* can be important vectors of *Anaplasma* in areas of enzootic instability, especially in dairy farms, feedlots and other intensive production systems, where the animals are very close to each other. The high density of animals favours the transmission of pathogens by horseflies due to their sensitive behaviour regarding the host's reaction and aggressive behaviour due to the need to ingest large amounts of blood for maturation of their oocytes^21,22^. This finding may also be important for addressing the geographic distribution of the species *D. missionum* and *P. lindneri* that coincide with areas of high production of beef and dairy cattle in South America. The species *P. lindneri* occurs in Argentina (Formosa, Chaco, Santa Fé, and Entre Ríos), Paraguay, Uruguay (Montevideo, Colonia, Paysandu and Tacuarembó)^15,23^ and Brazil (Mato Grosso do Sul and Rio Grande do Sul)^24^. *D. missionum* is one of the four most frequent species in Tacuarembó, Uruguay^15^, and is considered rare in collections from the coastal plain of Rio Grande do Sul, Pampa biome^25^. It was originally described from specimens collected during the Jesuit missions in Rio Grande do Sul, and its distribution extends to Argentina (Misiones, Buenos Aires, Santa Fé)^23^.

*A. marginale* was detected in only one individual each of *T. fuscofasciatus* and *T. tacuaremboensis*. *T. fuscofasciatus* has a wide distribution, including the Cerrado biome in the state of Goiás to Rio Grande do Sul in Brazil^23,25^, Uruguay (Tacuarembó and Paysandú)^15^, Bolivia, Argentina (Salta, Santa Fe, Formosa, Chaco, Entre Ríos, and Misiones) and Paraguay^23^. This species is less abundant than others^15,25^. *T. tacuaremboensis* was recently described^26^, and for now, this species is restricted to the Uruguayan Pampa.

The absence of positive detection in *T. campestris, T. claripennis* and *T.* aff. *platensis* may have been due to the low number of specimens used for each of these species to detect *A. marginale,* with the exception of *T. triangulum*. This species accounted for 22% of the specimens tested, showing no detection of the pathogen, despite being one of the most frequent species in NZI traps in the department of Colonia^15^. Recently, studies detected the presence of *Trypanosoma kaiowa*^27^ in 33% of specimens of *T. triangulum* in the coastal plain of Rio Grande do Sul and did not find other parasites of veterinary importance, despite the high abundance of this horsefly species in southern Rio Grande do Sul, Brazil^25,28^.

Although further studies are needed to determine the role of horseflies in the transmission of *A. marginale*^3^, especially in tick-free areas, these findings have significant implications for understanding the epidemiology of this important disease in South America.

## Methods

### Data collection

**Figure 2:**
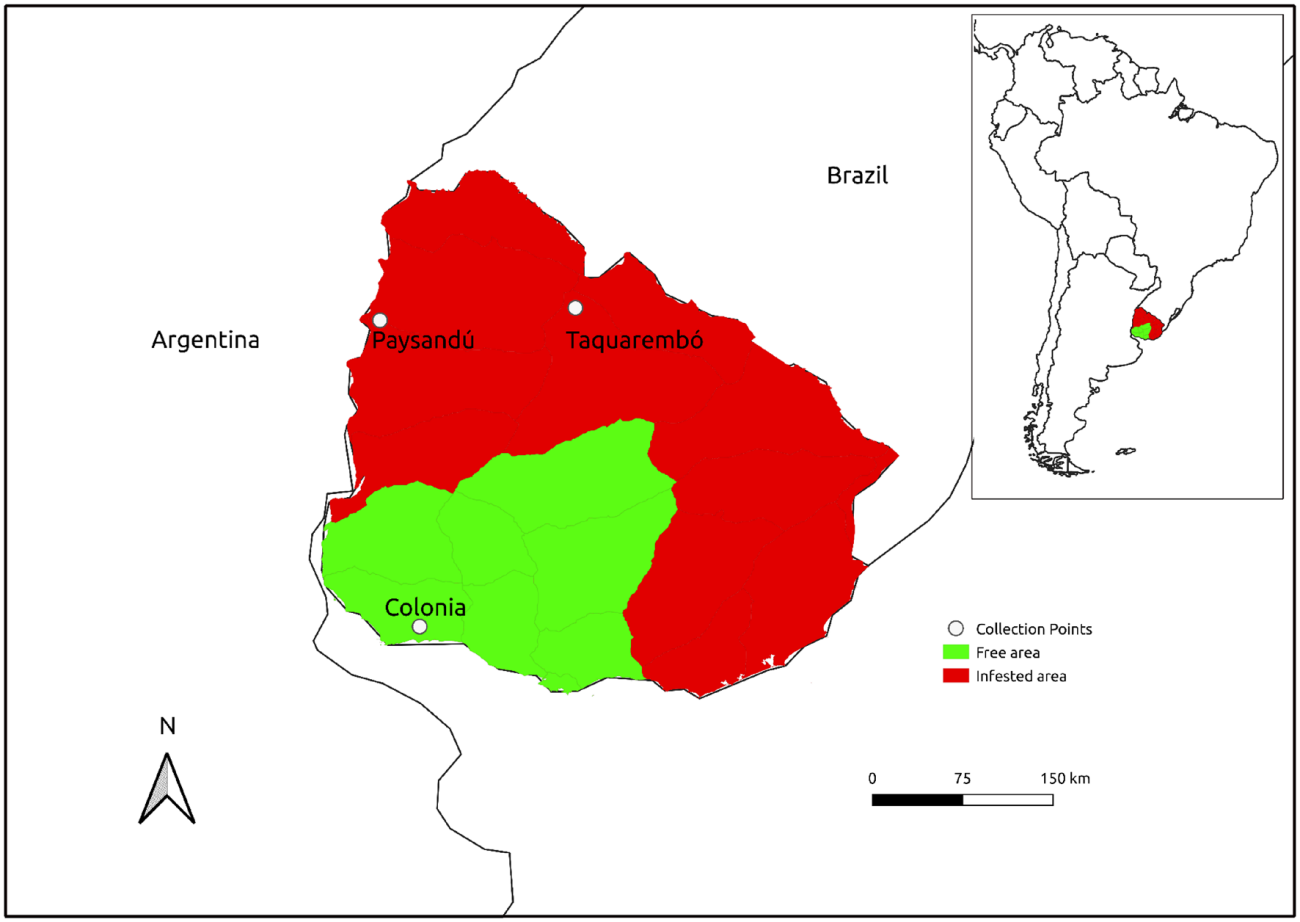
Locations of horsefly collections in tick-free and tick-infested areas of Uruguay. The map was created using QGIS 3.22.11 (http://qgis.org).

The collections were performed on farms located in the departments of Tacuarembó (three farms), Paysandú (one farm) and Colonia (two farms) and occurred between December 2017 and March 2019. These samplings were performed manually and/or with NZI traps. Using the manual sampling protocol, the horse flies were caught while feeding on animals. A defined collection time pattern was not established for the manual (feeding on cattle) or NZI trap^15^ method (Table 2, Fig. 2). The most abundant species were *Dasybasis missionum* (Macquart, 1838), *Poeciloderas lindneri* (Kröber, 1929), *Tabanus campestris* Brèthes, 1910, *Tabanus claripennis* Bigot, 1892, *Tabanus fuscofasciatus* Macquart, 1838, *Tabanus platensis* Brèthes, 1910, *Tabanus tacuaremboensis* Krolow, Lucas e Henriques, 2022 and *Tabanus triangulum* Wiedemann, 1828^15^.

**Table 2:** Location and capture method used for the nonsystematic horsefly collections in Uruguay.

Identifier	Location	Department	Capture method	Total N
M	31°21′37.8″S, 56°05′14.6″W	Tacuarembó	Manual (feeding on cattle)	8
M	31°28′29.4″S, 57°53′44.4″W	Paysandú	Manual (feeding on cattle)	4
NZI	31°28′29.4″S, 57°53′44.4″W	Paysandú	NZI trap	33
NZI	34°17′30.2″S, 57°37′41.4″W	Colonia	NZI trap	49
M	34°18′14.1″S, 57°31′42.7″W	Colonia	Manual (feeding on cattle)	4

### DNA extraction, PCR and sequencing

Total DNA extraction from whole Tabanidae individuals was performed using the PureLink^®^ Genomic DNA Mini Kit (Thermo Fisher Scientific Inc., USA). DNA concentration and quality were verified by spectrophotometry using a NanoVue™ Plus (GE Healthcare Life Sciences, USA), and only samples with absorption ratios in the range of 1.8–2.0 were subjected to PCR, following the same methodology used by Rodrigues et al.^28^.

The molecular detection of *A. marginale* was carried out using Major Surface Protein (MSP-5). This fragment is highly conserved for the genus and in all isolates of *A. marginale* and is widely used for the detection and confirmation of this species^29^. The primers used were forward (5′-GCATAGCCTCCGCGTCTTTC-3′) and reverse (5′-TCCTCGCCTTGGCCCTCAGA-3′), and the expected amplicon was 458 bp in length. The *A. marginale* DNA positive control was kindly supplied by F. Riet-Correa. The optimal PCR parameters were initial denaturation for 4 min at 94 °C, followed by 35 cycles of 94 °C for 1 min, 60 °C for 30 s, and 72 °C for 45 s, with a final extension of 4 min at 72 °C^30^.

The 25 µl PCR mixture consisted of 2 µl of DNA template (100 ng total input), 12.5 µl of GoTaq^®^ Green Master Mix 2 ×, 1 µl each of forward and reverse primer (10 µM each primer) and enough nuclease-free water to reach the total volume. Electrophoresis of PCR products was carried out at a constant voltage of 10 V/cm by using a Bio-Rad electrophoresis assembly with a 1.2% agarose gel in 0.5 × Tris Borate EDTA (TBE) buffer^28^. Products were purified by excluding unwanted components from the PCR using a PureLink^®^ PCR Purification Kit (Life Technologies, USA) following the manufacturer's instructions^28^.

Purified PCR products were quantified by using a UV spectrophotometer and inserted into the pCR™2.1-TOPO^®^ vector using the TOPO^®^ TA Cloning^®^ Kit (Life Technologies, USA). The cloning reaction with a 6 µl volume containing 4 µl of purified PCR products, 1 µl of salt solution and 1 µl of pCR™2.1-TOPO^®^ vector was incubated for 5 min at room temperature^28^. Each cloning reaction was used to transform electrocompetent *Escherichia coli* DH5α cells. The transformed cells were poured into Luria–Bertani agar (LB agar) plates with 100 mg/ml ampicillin and incubated overnight at 37 °C. The automatic sequencing of the positive clones was performed on a Biosystems 3500 Genetic Analyser^®^ (Life Technologies, USA) by using a Big Dye^®^ v3.1 Terminator Kit (Applied Biosystems, USA) (Life Technologies, USA)^28^.

### Phylogenetic analysis

A set of reference sequences was obtained by a BLAST search of the *msp5* sequence from *A. marginale* isolate GenEstrut against the GenBank database. Sequences with query coverage = 100%, a length > 350 bp, and available country information were selected. Additionally, *msp5* sequences from *Anaplasma ovis* (HM195102.1 and GQ483471.1) and *Anaplasma phagocytophilum* (EF185292.1 and EF185289.1) were retrieved from GenBank. The nucleotide sequences were aligned using the webPRANK webserver (https://www.ebi.ac.uk/goldman-srv/webprank/)^31^, followed by removal of ambiguous regions with Gblocks (http://phylogeny.lirmm.fr/)^32^. The phylogenetic analysis was performed on the IQ-TREE webserver (http://iqtree.cibiv.univie.ac.at/)^33^ using the ModelFinder application to select the best evolutionary model. Branch support was assessed using ultrafast bootstrap approximation (UFBoot) with 1000 replicates^34^, an approximate likelihood-ratio test based on a Shimodaira-Hasegawa-like procedure (SH-aLRT) with 1000 replicates^35^ and an approximate Bayes test.

## Data Availability

The sequences generated and/or analysed during the current study are available in the GenBank repository: https://www.ncbi.nlm.nih.gov/nuccore/OP047896.
